# Using B_4_C Nanoparticles to Enhance Thermal and Mechanical Response of Aluminum

**DOI:** 10.3390/ma10060621

**Published:** 2017-06-06

**Authors:** Fareeha Ubaid, Penchal Reddy Matli, Rana Abdul Shakoor, Gururaj Parande, Vyasaraj Manakari, Adel Mohamed Amer Mohamed, Manoj Gupta

**Affiliations:** 1 Center for Advanced Materials, Qatar University, Doha 2713, Qatar; fareeha.ubaid@qu.edu.qa (F.U.); penchal.matli@qu.edu.qa (P.R.M.); 2 Department of Metallurgical and Materials Engineering, Faculty of Petroleum and Mining Engineering, Suez University, Suez 43721, Egypt; gururaj.parande@u.nus.edu (G.P.); mbvyasaraj@u.nus.edu (V.M.); mpegm@nus.edu.sg (M.G.); 3 Department of Mechanical Engineering, National University of Singapore, Singapore 117576, Singapore; adel.mohamed25@yahoo.com

**Keywords:** Al-B_4_C nanocomposites, microwave sintering, hot extrusion, mechanical properties, thermal expansion

## Abstract

In this work, Al-B_4_C nanocomposites were produced by microwave sintering and followed by hot extrusion processes. The influence of ceramic reinforcement (B_4_C) nanoparticles on the physical, microstructural, mechanical, and thermal characteristics of the extruded Al-B_4_C nanocomposites was investigated. It was observed that the density decreased and porosity increased with an increase in B_4_C content in aluminum matrix. The porosity of the composites increased whereas density decreased with increasing B_4_C content. Electron microscopy analysis reveals the uniform distribution of B4C nanoparticles in the Al matrix. Mechanical characterization results revealed that hardness, elastic modulus, compression, and tensile strengths increased whereas ductility decreases with increasing B_4_C content. Al-1.0 vol. % B_4_C nanocomposite exhibited best hardness (135.56 Hv), Young’s modulus (88.63 GPa), and compression/tensile strength (524.67/194.41 MPa) among the materials investigated. Further, coefficient of thermal expansion (CTE) of composites gradually decreased with an increase in B_4_C content.

## 1. Introduction

Since the early 1990s, metal matrix composites (MMCs) have been the center of attention due to their high tensile strength, good thermal behavior, high thermal conductivity, high level of chemical inertness, and good wear resistance properties. These specific properties make them quite suitable for applications such as in aerospace, automobile, and electronics industries [1,2]. Aluminum and its alloys in this regard are of special interest because of their light weight, easy fabricability, superior mechanical properties, high stiffness, hardness, and corrosion response [3,4]. The ability to control the properties by optimization of volume percent, size, and reinforcing phase distribution makes hard ceramic reinforced metal matrix composites an interesting option [5,6,7,8]. Boron carbide (B_4_C) is being considered as a suitable reinforcement for MMCs due to its low density and identical mechanical and thermal properties as exhibited by SiC and Al_2_O_3_. The Al-B_4_C composites are used in bicycle frames, bullet proof vests, armor tanks, containment of nuclear waste, neutron absorbers in nuclear power plants, transportation applications, etc. owing to their high hardness, low density, and excellent thermal and chemical stability [9,10]. Recently, Al-B_4_C composites were prepared through different techniques: stir casting [11], squeeze casting [12], powder metallurgy method [13], spark plasma sintering [14], and microwave sintering [15]. However, in the making of MMCs, the selection of reinforcement particles depends on the application, manufacturing techniques, and cost of material. Both casting and powder metallurgy (PM) methods can be used to fabricate metal matrix nanocomposites. Historically, PM methods have been developed successfully and commercially used by different manufactures and have also been applied in the production of MMCs for aerospace applications. As compared to casting methods, PM approach has shown its advantage to produce uniform microstructures leading to developing high performance composite materials [16,17,18]. The development of metal matrix nanocomposites with light metal matrices are gaining increasing attention due to their attractive properties coupled with weight savings that can be realized for weight critical applications. These unique properties make them attractive for automotive and commercial applications at a reasonable cost. Nonetheless, to obtain the desired microstructure and improved mechanical properties the particles need to be homogeneously distributed in the matrix. Recent research has shown that energy efficient microwaves is a newly explored method and it has been applied successfully in processing of various materials, fabricating materials with improved mechanical properties. Microwave sintering is a distinguishing and alternative technique when compared with the existing processes utilizing orthodox heating sources [19,20] with a strong potential of enhanced turn-over and reduced energy consumption.

The aim of the present work was to fabricate high performance Al-B_4_C nanocomposites through a cost-effective processing technique based on PM route incorporating microwave sintering process followed by hot extrusion. The effect of reinforcement volume fraction on microstructure, physical, mechanical, and thermal characteristics of the composite have been examined.

## 2. Materials and Methods

Boron carbide (B_4_C) particles with an average diameter of ~10 nm (>99% purity, NaBond Tech., Shenzhen, China) was selected as reinforcement and high purity aluminum matrix (~7–15 μm, 99.9% purity, Alfa Aesar, Tewksbury, MA, USA) was used as the starting material to fabricate the Al-B_4_C metal matrix composites.

A mixture of Al powder with 0.5 vol. % and 1.0 vol. % of B_4_C was carefully weighed and mixed at room temperature using a Retsch PM400 planetary ball mill for 2 h with the milling speed of 200 rpm in order to get a homogeneous particle distribution. No balls were used in this stage. The blended powders were compacted into cylindrical billets (35 mm diameter and ~40 mm height) in a die at a pressure of 97 bar (50 ton) using a 100-ton hydraulic press under ambient conditions. Pure aluminum was compacted using the same parameters without blending. Finally, the compacted cylindrical billets were sintered using microwave sintering technique [5,21]. The billets were heated to 550 °C in a 900 W, 2.45 GHz SHARP microwave oven. Sintering of the green compacts was accomplished using a two-directional heating arrangement. Two-directional or hybrid heating is achieved through the use of microwaves and an external microwave susceptor (SiC) that couples with the microwaves readily thereby generating radiant heat. Microwaves heat the billet from within while the radiant heat from the susceptor heats the billet from the surface inwards. After heating, the compacts were left to cool naturally to near room temperature in the sintering setup before removal.

**Figure 1:**
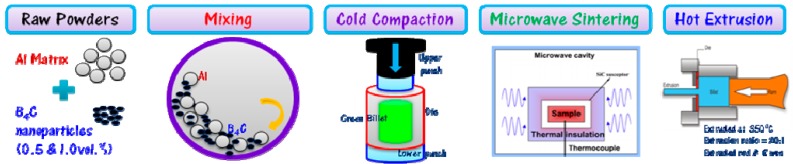
Schematic representation of microwave-hot extruded Al-B_4_C nanocomposites.

Prior to hot extrusion, billets of microwave sintered pure Al and its nanocomposites were soaked at 400 °C for 1 h and then subjected to thermo-mechanical treatment (hot extrusion) at 350 °C and 500 MPa as a secondary processing. The extrusion ratio was about 20.25:1 to produce an 8-mm diameter extruded rod and approximately 350 mm long. Colloidal graphite was used as lubricant during extrusion. After extrusion, the extruded rods were air cooled to room temperature. These extruded rods were subsequently used for characterization studies. The schematic of experimental methodology to fabricate Al-B_4_C nanocomposites is shown in Figure 1. 

The Archimedes principle was used to determine the density of extruded Al-B_4_C nanocomposite samples. The porosity of extruded Al composites was calculated by using the following relation

(1) $$\rho =\frac{\rho_{th}-\rho_{\exp}}{\rho_{th}-\rho_{air}}\times 100$$

where, ρ_*th*_, ρ_exp_, and ρ_*air*_ are the theoretical, experimental, and air density in (g/cm^3^), respectively.

The phase identification of the extruded samples was carried out using X-ray powder diffractometer (PANalytical X’pert Pro, PANalytical B.V., Almelo, The Netherlands) based on Cu-K_α_ radiation (1.541 Å) in the 2θ range of 30–80° at scan rate of 0.2°/min.

Field emission scanning electron microscopy (Hitachi FESEM-S4300, Tokyo, Japan) with energy dispersion spectroscopy (EDS) was used to identify the reinforcement phase and microstructure of the extruded nanocomposite samples.

Coefficient of thermal expansion (CTE) of Al-B_4_C nanocomposites were determined using thermo-mechanical analyzer (INSEIS TMA PT 1000LT, Linseis Thermal Analysis, NJ, USA). A heating rate of 5 °C/min for a temperature range of 50–350 °C with argon flow rate of 0.1 lpm was used for the experiment.

The hardness testing of the pure Al and nanocomposite samples was carried out using Vicker’s hardness tester with applied load of 0.1 kgf for 15 s as per the ASTM standard E384-08 [22]. Nanoindentation analysis was performed using a MFP-3D Nano Indenter (head connected to AFM equipment, Asylum Research, Buckinghamshire, UK) system equipped with standard Berkovich diamond indenter tip. The testing was performed at room temperature. The applied forces are in the mN range, and penetration depths from several nm to μm are used to compute the hardness (*H*) and Young’s modulus (*E*). The indentation was made to a maximum load of about 100 mN and under loading and unloading rate of 200 μN/s and dwell time at maximum load 5 s.

Compressive testing of the cylindrical specimens was performed at room temperature according to ASTM E9-89a [23] using a universal testing machine (Lloyd Instruments Ltd., Sussex, UK). The test specimens with a length to diameter (l/d) ratio ~1 were subjected to a compression load at a constant strain rate of 8.3 × 10^−4^/s. From the load displacement curves, the ultimate compression strength (MPa), yield strength, and failure stain were measured.

Tensile tests were carried out on pure Al and nanocomposite samples according to ASTM E8/E8M-15a [24] on universal testing machine at room temperature with the tensile rate of 8.3 × 10^−4^/s. From the load displacement curves, 0.2% offset yield strength (YS), ultimate tensile strength (UTS), and elongation (ductility) were determined.

## 3. Results and Discussion

**Figure 2:**
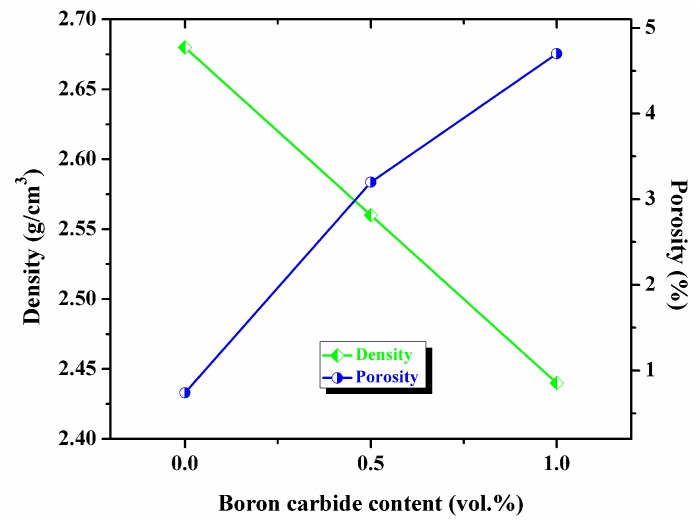
The variation of density and porosity of microwave-hot extruded Al-B_4_C nanocomposites.

The variation of density and porosity of microwave-hot extruded Al-B_4_C nanocomposites with respect to increasing B_4_C content are shown in Figure 2. It can be observed that the density values decrease with increasing reinforced ceramic particles while the porosity is increases. Since the density of boron carbide (2.52 g/cm^3^) is less than the density of pure Al (2.70 g/cm^3^), the overall density of the Al-B_4_C nanocomposites is reduced by increasing amount of porosity. Changes in the B_4_C content causes a higher amount of porosity formed in composites. Therefore, the density of Al-0.5 vol. % B_4_C nanocomposites is higher than the density of Al-1.0 vol. % B_4_C composites. These results are also supported with the results already reported by Busquets et al. [25].

**Figure 3:**
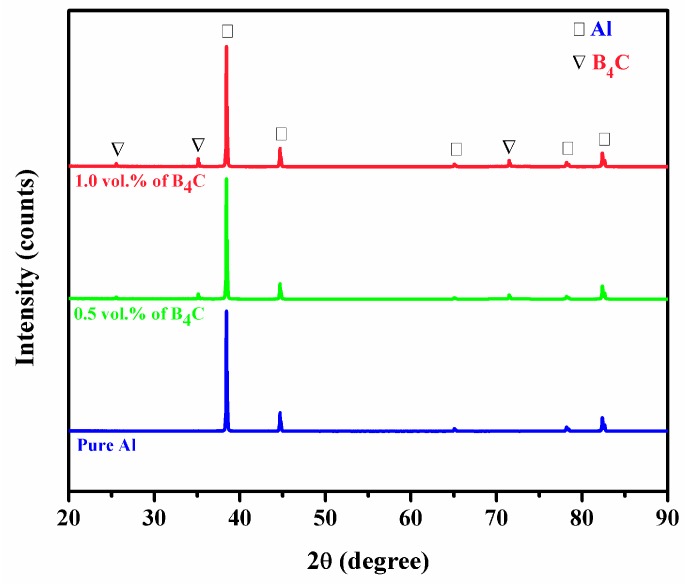
XRD patterns of microwave-hot extruded Al-B_4_C nanocomposites.

Figure 3 shows the XRD analysis of extruded pure Al and Al-B_4_C nanocomposites. The XRD patterns reveal prominent characteristic crystalline peaks of Al and rhombohedral B_4_C [26]. The XRD pattern indicated that the peak intensity increases with increasing percentage of B_4_C nanoparticles.

**Figure 4:**
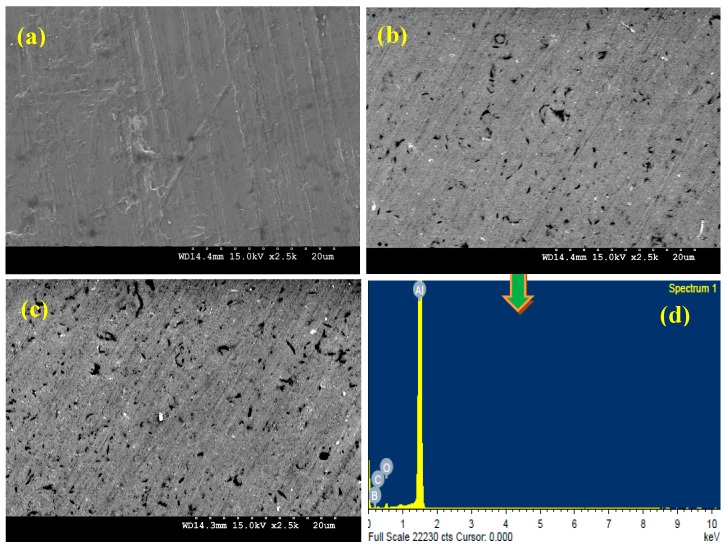
SEM micrographs (**a**–**c**) and EDS spectrum (**d**) of microwave-hot extruded of Al-B_4_C nanocomposites.

One of the primary objectives of the present research work is to ensure homogenous distribution of B_4_C particles in the aluminum matrix, as for Al-MMCs, good mechanical performance depends strongly on a homogenous distribution of the reinforcement in the final product [27,28,29]. Scanning electron microscopy (SEM) was used in order to study the microstructure of the developed nanocomposites. Figure 4a–c shows the typical microstructures of the composites reinforced with 0 vol. %, 0.5 vol. % and 1.0 vol. % B_4_C nanoparticles, respectively. It can be further noted that SEM images show two main phases; the grey matrix is the Al phase while the dispersed phase showing white spots represents the B_4_C nanoparticles. Increasing amount of B_4_C in the matrix results in an increase in the internal porosity. This increase in the porosity can be ascribed to the poor wettability between aluminum and B_4_C. However, these agglomerated sites are only observed in few locations through the matrix and a near-uniform nanoparticles distribution is noticed in the Al-B_4_C nanocomposite samples. This near-uniform distribution of nanoparticles promotes more uniform heating through microwaves and demonstrates the effectiveness of using hybrid microwave sintering for the synthesis of Al-based nanocomposites [30].

Hence, the SEM results show that microwave sintering followed by hot extrusion has good potential for synthesizing the particulate reinforced metal matrix composites because this method can produce a composite with good reinforcement dispersion and acceptable levels of porosity. The Energy Dispersive Spectroscopy (EDS) analysis showed the presence of Al and B_4_C phases. The EDS analysis spectrum of Al-0.5 vol. % B_4_C nanocomposite is presented in Figure 4d.

**Figure 5:**
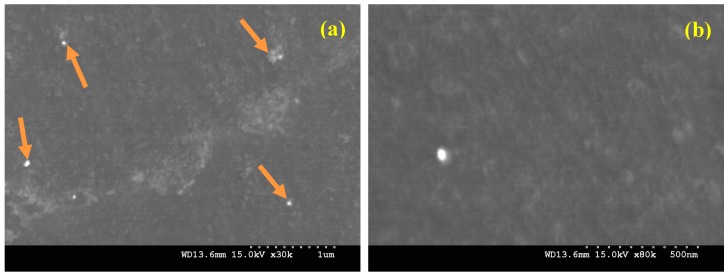
(**a**) Distribution of B_4_C nanoparticles and (**b**) interfacial integrity of Al-1.0 vol. % B_4_C nanocomposite.

Further, from the micrographs (Figure 5) representing the distribution of B_4_C nanoparticles within the synthesized Al-1.0 vol. % B_4_C nanocomposites, minimal agglomeration of B_4_C particulates was observed.

**Figure 6:**
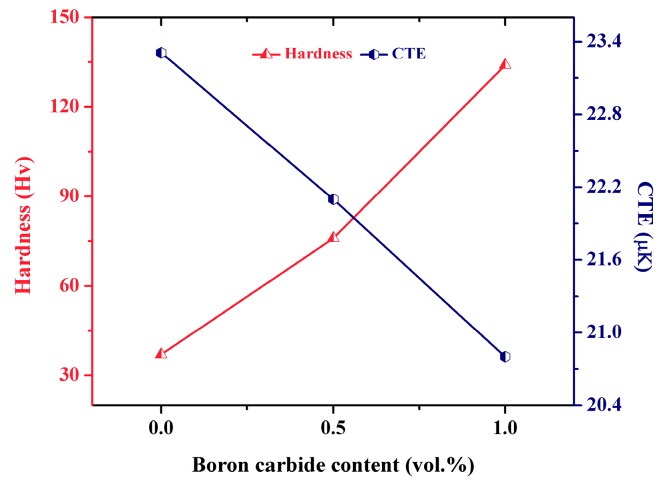
Hardness and CTE of microwave-hot extruded Al-B_4_C nanocomposites.

The average microhardness of the Al-B_4_C nanocomposites is shown in Figure 6. It can be seen that the hardness of the Al-B_4_C composites enhanced with increasing the B_4_C content in the Al matrix. The average hardness values of the Al-0.5 vol. % B_4_C and Al-1.0 vol. % B_4_C nanocomposites are measured to be 78 ± 5 and 135 ± 3 Hv respectively, which are much higher than the pure Al, i.e., 37 ± 6 Hv. The increase in the microhardness of AMMCs indicates that the ceramic particles have a major contribution in the strengthening of Al matrix. This increase in the hardness is because of the contribution of the reduced crystallite size of ~10 nm (B_4_C) in the composite and uniform distribution of extremely harder boron carbide nanoparticles [31,32].

The presence of hard ceramic particles can enhance the microhardness of composites according to the rule of mixtures [33].

(2) *H*_c_ = *H*_m_*f*_m_ + *H*_r_*f*_r_

where *H*_c_ represents hardness of the composite, *H*_m_ and *H*_r_ represent hardness of the matrix and the reinforcing particle, respectively, and *f*_m_ and *f*_r_ represent the volume fraction of the matrix and the reinforcing particle, respectively.

Figure 6 shows the variation of the coefficient of thermal expansion (CTE) of Al-B_4_C nanocomposites as a function of B_4_C volume fraction. Generally, the CTE values decreases with the addition of B_4_C content. The reduction in the coefficient of thermal expansion (CTE) of composite samples can be attributed to the presence of nano B_4_C reinforcements which has lower CTE value (3.2 × 10^−6^ K^−1^) [34] when compared to pure Al (24 × 10^−6^ K^−1^) [35]. The CTE of pure Al was measured to be 23.31 × 10^−6^ K^−1^ which is in close agreement with the theoretical CTE of aluminum (24 × 10^−6^ K^−1^). The addition of nano-sized 1.0 vol. % B_4_C nanoparticles to Al reduced the CTE value to ~20.03 × 10^−6^ K^−1^ which is ~14% reduction when compared to pure Al. In addition, the linear decrease in CTE values with the addition of ceramic nanoparticles can be attributed to: (a) the lower CTE values of ceramic (B_4_C) nanoparticle reinforcements as compared to that of the pure Al matrix; (b) uniform distribution of the ceramic reinforcements in the matrix.

The hardness and Young’s modulus of Al-B_4_C nanocomposites data are presented in Table 1. The results indicate that both the hardness and Young’s modulus of the Al-B_4_C nanocomposites have been improved significantly. For instance, the Young’s modulus was improved from 73.19 GPa to 88.63 GPa by increasing the content of nano-size B_4_C from 0 vol. % to 1.0 vol. %. It is noted that the Young’s modulus of microwave-hot extruded Al-B_4_C nanocomposites is higher than that of conventionally sintered Al-B_4_C composites [36]. The improvement in the hardness and Young’s modulus of the composites can be mainly attributed to: (a) B_4_C has a Young’s modulus as high as 400 GPa [37] and (b) the dispersion strengthening effect due to uniform distribution of B_4_C nanoparticles in the matrix [38].

**Figure 7:**
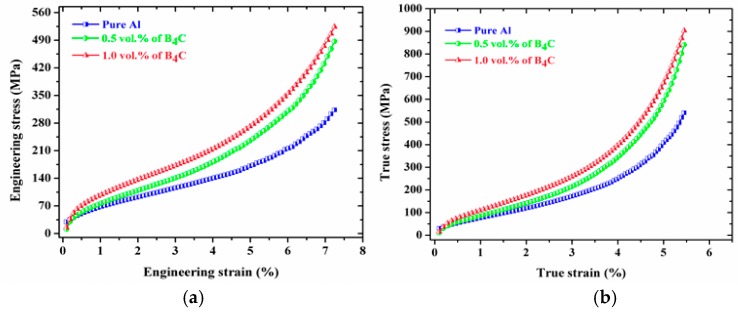
Compression engineering stress–strain curves (**a**) and compression true stress-strain curves (**b**) of the microwave-hot extruded Al-B_4_C nanocomposites.

The engineering and true stress-strain curves of the Al-B_4_C nanocomposites under compression loading at room temperature are shown in Figure 7a,b. The average compressive yield strength (CYS) and ultimate compressive strength (UCS) values of the extruded composites are listed in Table 1. A significant improvement in the strength of Al-B_4_C nanocomposites are observed compared to pure aluminum. The compression strength of pure Al was improved by adding various ceramic reinforcement particles. The Al-1.0 vol. % B_4_C nanocomposite showed compressive yield strength (0.2% CYS) and ultimate compressive strength (UCS) of ~124.24 MPa and ~524.67 MPa, respectively, the incremental increase is ~57% and ~67.5% respectively compared to pure Al. The improvement in the compressive strength of the Al-B_4_C nanocomposites compared to pure Al can be ascribed to the uniform distribution of reinforcing nanoparticles in the matrix and enhanced dislocation density [39].

**Figure 8:**
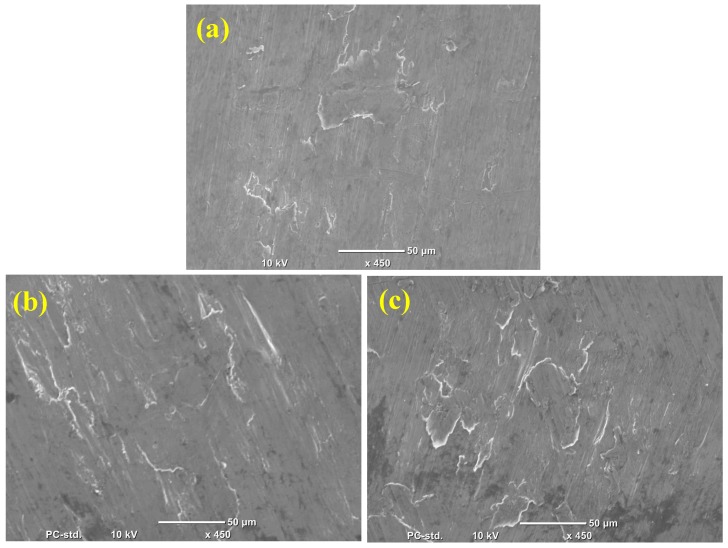
Compression fracture images of: (**a**) Pure Al (**b**) Al-0.5 vol. % B_4_C and (**c**) Al-1.0 vol. % B_4_C nanocomposite.

The fracture morphology of microwave sintered-extruded pure Al and Al-B_4_C nanocomposites during compression tests are shown in Figure 8a–c. The fracture surfaces are comparatively smooth and the formation of shear band is not evident in the fractured samples. It approves that the compressive deformation of the Al-composites is expressively indifferent. This is due to heterogeneous deformation and work hardening behavior.

**Figure 9:**
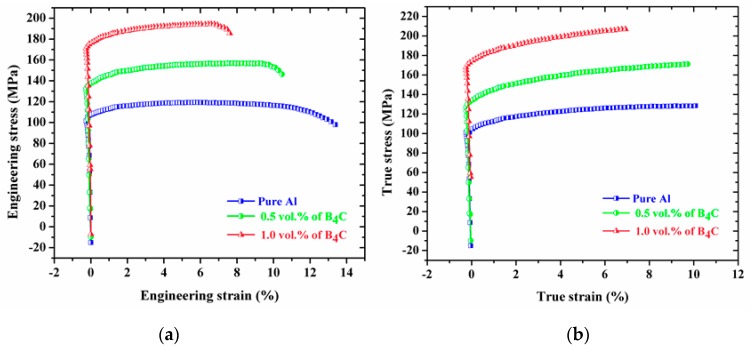
Tensile engineering stress-strain curves (**a**) and tensile true stress-strain curves (**b**) of the microwave-hot extruded Al-B_4_C nanocomposites.

The engineering and true stress–strain curves of the Al-B_4_C nanocomposites under tensile loading at room temperature are shown in Figure 9a,b. It indicates that all extruded composites exhibited higher tensile strength in comparison to that of pure Al. The results show that considerable increment in tensile yield strength (TYS) and ultimate tensile strength (UTS) of Al nanocomposites were obtained due to presence of hard ceramic reinforcing particles. When compared to pure Al, the developed Al-1.0 vol. % B_4_C nanocomposites showed enhancement in TYS (from 105.12 MPa to 173.14 MPa) and UTS (from 116.41 MPa to 194.41 MPa) values. Increasing volume fraction of B_4_C nanoparticles leads to decrease in ductility of the metal matrix composites due to the particle agglomeration and porosity. As mentioned, an increase in the percentage of B_4_C nanoparticles increases the amount of porosity; consequently, the composite ductility decreases. Therefore, the ductility of the 1.0 vol. % B_4_C nanocomposite is lower than that of the 0.5 vol. % B_4_C nanocomposite and the pure Al.

With the addition of B_4_C nanoparticles, the strength properties (compressive and tensile) are found to improve with respect to B_4_C volume fraction. In any metal matrix composite, the increase in strength when compared to pure aluminum can be attributed to the following reasons [40,41,42,43]: (i) active load transfer from the matrix to the reinforcement; (ii) Orowan strengthening; and (iii) generation of internal thermal stresses because of the difference in the co-efficient of thermal expansion (CTE) between the reinforcement particles and matrix phase.

The efficient load transfer (σ_*load*_) between the ductile matrix and the hard-ceramic reinforcement particles during tensile testing occurs, particularly when there is a good interfacial contact between the matrix and the reinforcement and is represented as

(3) $$\sigma_{load}=0.5V_{f}\sigma_{YM}$$

where, *V_f_* is the volume fraction of ceramic reinforcement particles and σ_*YM*_ is the matrix yield stress.

The interaction between the dislocations and the reinforcement particles enhances the strength of the composite materials in agreement with the Orowan mechanism. Due to the existence of dispersed reinforcement particles in the matrix, dislocation loops are formed when dislocations interact with the reinforcing particles. σ_*Orowan*_ can be calculated as

(4) $$\sigma_{Orowan}=\frac{0.13Gb}{\lambda }\ln\frac{r}{b}$$

where, *G* is the shear modulus of matrix, *b* is the Burgers vector, λ is the inter-particle spacing, and *r* is the particle radius.

The difference in the CTE values of the reinforcement particles and the metal matrix produces geometrically necessary dislocations and thermally induced residual stresses. The thermal stresses at the particles and matrix interface make the plastic deformation more tough which, hence, enhances the level of hardness and flow stress. The mismatch strain effect due to the difference between the CTE values of particles and that of the matrix is given by

(5) $$\Delta \sigma_{CTE}=\sqrt{3}\beta G_{m}b\sqrt{\frac{24V_{f}\Delta \alpha \Delta T}{(1-V_{f})br_{p}}}$$

where, *b* is the strengthening coefficient, Δα is the difference between CTE of matrix and reinforcement, and Δ*T* is the difference between the test and process temperature.

**Figure 10:**
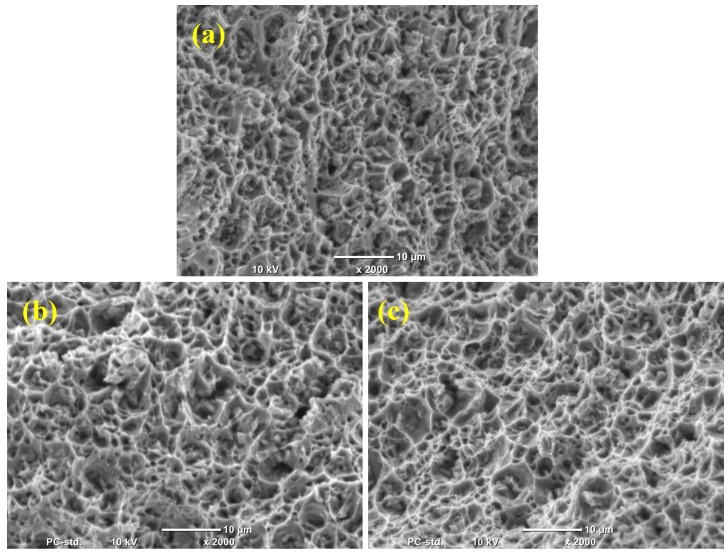
Tensile fracture images of: (**a**) Pure Al (**b**) Al-0.5 vol. % B_4_C and (**c**) Al-1.0 vol. % B_4_C nanocomposite.

The tensile fracture surfaces of pure Al and Al-B_4_C nanocomposites are shown in Figure 10. The examination of fractured surfaces reveals the formation of similar ductile fracture in all composites. A large number of dimples with tear ridges is also seen in the Al-B_4_C nanocomposite.

The dimples are smaller in size and have shallow depth which gives the impression of dominant ductile fracture, which explains the higher ultimate stress and total elongation values in Al-B_4_C composites. The surface morphology of fractures of nanocomposites with B_4_C reinforcement produced with the microwave sintering method is identical to that of the B_4_C reinforced Al matrix composites material produced by powder metallurgy followed by hot extrusion by Gomez et al. [25].

## 4. Conclusions

Pure Al and Al-B_4_C (0 vol. %, 0.5 vol. %, and 1.0 vol. %) nanocomposites were successfully synthesized using microwave sintering approach followed by hot extrusion. The following conclusions can be drawn:

- The density of Al nanocomposites decreases, whereas the porosity increases with an increase in volume percentage of the reinforcement;
- The microstructural studies revealed the uniform distribution of the B_4_C particles in the Al matrix;
- Hardness of the Al-B_4_C nanocomposite increased with an increase in the amount of B_4_C particles;
- The CTE of the Al nanocomposites decreases with the increase in percentage of the reinforcement;
- The increasing presence of nano-sized B_4_C particulate leads to an increase in YS and UCS;
- The addition of hard B_4_C nanoparticles in pure aluminum led to an increase in both YS and UTS, but ductility behavior showed a reverse trend;
- The shear band and dimple formations were observed in Al nanocomposites under compression and tensile loading, respectively.

## Figures and Tables

**Table 1:** Mechanical properties of pure Al and Al-B_4_C nanocomposites

Sample	Hardness		Young’s Modulus (GPa)	Compressive Properties		Tensile Properties		
	(Hv)	(GPa)		CYS (MPa)	UCS(MPa)	TYS (MPa)	UTS (MPa)	Elongation (%)
Pure Al	37.14	5.15	73.19	79.10	313.63	105.12	116.41	13.6
Al-0.5 vol. % B_4_C	78.85	9.60	78.52	98.56	482.54	132.68	156.90	10.6
Al-1.0 vol. % B_4_C	135.56	17.44	88.63	124.24	524.67	173.14	194.41	7.7
